# Cardiac computed tomography-derived epicardial fat volume and attenuation independently distinguish patients with and without myocardial infarction

**DOI:** 10.1371/journal.pone.0183514

**Published:** 2017-08-24

**Authors:** Amir Abbas Mahabadi, Bastian Balcer, Iryna Dykun, Michael Forsting, Thomas Schlosser, Gerd Heusch, Tienush Rassaf

**Affiliations:** 1 West German Heart and Vascular Center Essen, Department of Cardiology and Vascular Medicine, University Clinic Essen, Essen, Germany; 2 The Department of Diagnostic and Interventional Radiology and Neuroradiology, University Clinic Essen, Essen, Germany; 3 The Institute for Pathophysiology, University Clinic Essen, Essen, Germany; Klinikum Region Hannover GmbH, GERMANY

## Abstract

**Background and objective:**

Epicardial adipose tissue (EAT) volume is associated with coronary plaque burden and adverse events. We aimed to determine, whether CT-derived EAT attenuation in addition to EAT volume distinguishes patients with and without myocardial infarction.

**Methods and results:**

In 94 patients with confirmed or suspected coronary artery disease (aged 66.9±14.7years, 61%male) undergoing cardiac CT imaging as part of clinical workup, EAT volume was retrospectively quantified from non-contrast cardiac CT by delineation of the pericardium in axial images. Mean attenuation of all pixels from EAT volume was calculated. Patients with type-I myocardial infarction (n = 28) had higher EAT volume (132.9 ± 111.9ml vs. 109.7 ± 94.6ml, p = 0.07) and CT-attenuation (-86.8 ± 5.8HU vs. -89.0 ± 3.7HU, p = 0.03) than patients without type-I myocardial infarction, while EAT volume and attenuation were only modestly inversely correlated (r = -0.24, p = 0.02). EAT volume increased per standard deviation of age (18.2 [6.2–30.2] ml, p = 0.003), BMI (29.3 [18.4–40.2] ml, p<0.0001), and with presence of diabetes (44.5 [16.7–72.3] ml, p = 0.0002), while attenuation was higher in patients with lipid-lowering therapy (2.34 [0.08–4.61] HU, p = 0.04). In a model containing volume and attenuation, both measures of EAT were independently associated with the occurrence of type-I myocardial infarction (OR [95% CI]: 1.79 [1.10–2.94], p = 0.02 for volume, 2.04 [1.18–3.53], p = 0.01 for attenuation). Effect sizes remained stable for EAT attenuation after adjustment for risk factors (1.44 [0.77–2.68], p = 0.26 for volume; 1.93 [1.11–3.39], p = 0.02 for attenuation).

**Conclusion:**

CT-derived EAT attenuation, in addition to volume, distinguishes patients with vs. without myocardial infarction and is increased in patients with lipid-lowering therapy. Our results suggest that assessment of EAT attenuation could render complementary information to EAT volume regarding coronary risk burden.

## Introduction

Epicardial adipose tissue (EAT) volume is associated with coronary artery plaque burden and the prevalence and incidence of myocardial infarction.[1–6] In subjects with subsequent myocardial infarction, the fat volume surrounding the coronary segment developing the culprit lesion years later was even further increased compared to other segments in the same patients, suggesting that changes in adipose tissue locally influence plaque development.[3] In addition to volume, CT-derived fat attenuation is suggested to reflect unfavorable metabolic activity, as it increases with vascularization, reflects higher concentration of mitochondria and is correlated with local and systemic inflammatory markers.[7–9] Peri-coronary fat attenuation varies depending on its location,[10] however, clinical implications of EAT attenuation remain controversial.[9, 11] In the present manuscript, we aimed to (1) determine the distribution of CT-derived EAT volume and attenuation as well as their correlation in a retrospective clinical cohort of patients undergoing cardiac CT imaging, (2) investigate the association of EAT volume and attenuation with established risk factors as well as antihypertensive and lipid-lowering therapy, and (3) determine the association of EAT volume and attenuation with the clinical presentation of the patients.

## Materials and methods

### Study sample

We retrospectively included consecutive patients with myocardial infarction who received both cardiac CT and invasive angiography as part of clinical workup during index hospitalization between 2010 and 2015 (n = 50). Indication for CT imaging was heterogeneous and made for clinical reasons. Indications included CTA for suspected CAD in patients with low- to intermediate pretest likelihood for presence of CAD and evaluation of coronary anatomy, quantification of coronary plaque burden, evaluation of aortic aneurysms, or other cardiac structures after invasive coronary angiography. Median time between revascularization and CT was 7 days, while only 6 patients received the CT imaging prior to invasive angiography. Indications for CT imaging were heterogeneous and included quantification of coronary plaque burden, evaluation of aortic aneurysms and others. In addition, 50 randomly selected patients with stable coronary artery disease and without acute coronary syndrome, also receiving cardiac CT imaging during clinical workup, were included in the analysis. Patients with prior bypass surgery or other open heart surgery were excluded from our analysis. In addition, CT image quality not sufficient for further analysis or lack in clinical information from available hospital records, not allowing the assessment of risk factor profile or evaluation of clinical course of the patients, resulted in exclusion from our analysis (n = 6, 4 with myocardial infarction, 2 without myocardial infarction). The study protocol conforms to the ethical guidelines of the 1975 Declaration of Helsinki as reflected in a priori written approval by the institutional ethics committee of the University Clinic Essen. All data were fully anonymized before access by the researchers.

### CT-Imaging and fat quantification

CT imaging was performed using dual source computed tomography (Siemens Somatom Definition Flash or Somatom Force, Siemens, Forchheim, Germany). A non-contrast enhanced CT-scan for the assessment of coronary artery calcification, prospectively triggered at 70% of the RR-interval with 3mm slice thickness, was followed by CT angiography. Since the present data are based on a retrospective analysis, scan parameters differed based on scanner type and patient characteristics. CT examinations were performed using prospective triggering in most cases. Tube voltage was adjusted based on patients habitus.

From non-contrast CT images, EAT volume and attenuation were quantified offline using a dedicated workstation (Aquarius Workstation, TeraRecon, Foster City, CA, USA) as previously described.[5] In brief, EAT volume was assessed for all patients by manual tracing of the pericardial sac as outer border in axial planes from the right pulmonary artery to the apex of the heart. Within the region of interest, fat was defined as pixels within a window of -195 to -45 HU and a window center of -120 HU.[12] After 3-dimensional reconstruction, fat volume was automatically calculated by the software program. EAT attenuation was calculated as mean Houndsfield units of all pixels, which were counted as EAT volume. All EAT measures were performed by a single reader.

### Risk factor and clinical assessment

Assessment of risk factors and clinical diagnosis of patients were performed from all available hospital records. Systolic and diastolic blood pressure was obtained from admission records. Elevated blood pressure was defined as systolic blood pressure >140mmHg or diastolic blood pressure of >90mmHg. Total, HDL, and LDL-cholesterol as assessed within the same hospital stay were recorded. Diabetes was defined based on fasting glucose levels, HbA1c levels, and medication.[13] Active smoking and positive family history of premature coronary artery disease were assessed as documented by the treating physicians. Likewise, patients were regarded as having a myocardial infarction or stable coronary artery disease based on all available hospital records including lab results, ECGs, discharge letters and coronary angiography reports. Further, within the group of patients with myocardial infarction, we stratified by coronary or non-coronary cause of infarction, as defined as type-I or type-II myocardial infarction according to the current ESC definition.[14]

### Statistical analysis

Continuous variables are reported as mean ± standard deviation (SD). Discrete variables are given in frequency and percentiles. EAT volume and CT-attenuation were normally distributed. The Pearson correlation coefficient was used to determine the correlation of EAT volume with attenuation. The association of EAT volume and attenuation with risk factors was assessed by linear regression analysis. The following adjustment sets were used: model 1: unadjusted; model 2: adjusting for age, gender, BMI, systolic blood pressure, antihypertensive medication, LDL- and HDL-cholesterol, lipid-lowering medication, diabetes, smoking, positive family history. For continuous variables, standardized beta estimates were calculated per 1 standard deviation change of risk factor. EAT volume and attenuation in patients with and without myocardial infarction were compared using two-sided t-test. The association of EAT volume and attenuation with any myocardial infarction was assessed using logistic regression analysis. In separate analysis, associations were calculated comparing patients with and without type-I myocardial infarction. Odds ratios (OR) and 95% confidence intervals (CI) were calculated per each standard deviation change of EAT volume / attenuation. Since the overall number of myocardial infarctions was limited, adjustment was restricted to the following models: model 1: adjusting for EAT attenuation / volume; model 2: adjusting for EAT attenuation / volume, age, and gender; model 3: ancillary adjusting for BMI and lipid-lowering medication. Frequencies of myocardial infarction were further calculated in groups of patients with combination of EAT volume and attenuation above and below the median. All analyses were performed using SAS software (Version 9.4, SAS Institute Inc.). A p-value of <0.05 indicated statistical significance.

## Results

Overall, 94 patients (mean age 66.9 years (SD 14.7 years), 61% male) were included in our analysis with detailed patient characteristics depicted in Table 1. While the majority of patients were under antihypertensive medication (91%), 40% of the subjects still had elevated blood pressure. Likewise, the rate of patients with lipid lowering therapy was high (80%). Overall, 23% of patients were diabetics, while only 13% were active smokers. Both EAT volume and CT-derived attenuation were normally distributed, but the volume had wider range (IQR: 67.0ml; 144.6ml) than the attenuation (IQR: -91.4HU; -85.6HU). Likewise, the low standard deviation of CT derived attenuation reflected its low variation compared to EAT volume (standard deviation: 4.5 HU for attenuation, 60.1 ml for volume). EAT volume and attenuation showed only modest, but significant inverse correlation (r = -0.24, p = 0.02, S1 Fig).

**Table 1:** Patient characteristics.

	N = 94	
Age (years)	66.9, SD 14.7	
Gender (% male)		57 (60.6)
BMI (kg/m^2^)		27.0, SD 4.8
Systolic blood pressure (mmHg)		139.8, SD 26.4
Diastolic blood pressure (mmHg)		75.0, SD 14.7
Antihypertensive medication (%)		86 (91.5)
Total cholesterol (mg/dl)		183.2, SD 42.6
LDL cholesterol (mg/dl)		111.3, SD 38.1
HDL cholesterol (mg/dl)		51.2, SD 18.6
Lipid-lowering medication (%)		75 (79.8)
Diabetes (%)		22 (23.4)
Active smoking (%)		12 (12.8)
Positive family history (%)		31 (33.0)
EAT volume (ml)	116.6, SD 60.1	
EAT attenuation (HU)	-88.3, SD 4.5	

### Association of EAT volume and attenuation with risk factors

In univariate regression analysis, EAT volume was associated with age, BMI, and diabetes, while a trend was observed for male gender and HDL-cholesterol (Table 2). In multivariate regression analysis, age, gender, and BMI remained independently associated with EAT volume. In contrast, CT-derived EAT attenuation was higher in patients under lipid-lowering therapy. Upon adjustment for all risk factors, effect sizes for the association of lipid-lowering therapy with EAT attenuation remained stable, however, did not reach statistical significance (Table 2).

**Table 2:** Association of EAT volume and CT-attenuation with traditional cardiovascular risk factors in unadjusted and risk factor adjusted linear regression analysis.

	EAT volume				EAT attenuation			
	unadjusted		MV adjusted		unadjusted		MV adjusted	
	Beta estimate	p-value	Beta estimate	p-value	Beta estimate	p-value	Beta estimate	p-value
Age	18.2 (6.2–30.2)	0.003	28.5 (16.8–40.2)	<0.0001	-0.47 (-1.42–0.48)	0.3	-0.91 (-2.11–0.28)	0.1
Male Gender	24.6 (-0.2–49.5)	0.052	22.7 (0.5–45.0)	0.046	0.54 (-2.45–1.36)	0.6	-0.67 (-2.95–1.60)	0.6
BMI	29.3 (18.4–40.2)	<0.0001	29.9 (18.8–41.1)	<0.0001	0.06 (-0.88–1.00)	0.9	-0.21 (-1.35–0.92)	0.7
Systolic blood pressure	1.5 (-11.1–14.1)	0.8	-8.3 (-19.5–3.0)	0.1	0.01 (-0.95–0.97)	1.0	0.40 (-0.74–1.55)	0.5
Antihypertensive medication	25.1 (-18.9–69.2)	0.3	1.2 (-39.4–41.8)	1.0	0.03 (-3.32–3.37)	1.0	-0.50 (-4.65–3.64)	0.8
LDL cholesterol	-4.3 (-16.9–8.3)	0.5	5.4 (-5.1–15.9)	0.3	0.34 (-0.60–1.30)	0.5	-0.04 (-1.11–1.03)	0.9
HDL cholesterol	-11.8 (-24.2–0.6)	0.06	-7.6 (-18.8–3.7)	0.2	-0.38 (-1.33–0.56)	0.4	-0.42 (-1.56–0.73)	0.5
Lipid-lowering medication	-7.1 (-37.9–23.7)	0.6	-5.4 (-30.9–20.2)	0.7	2.34 (0.08–4.61)	0.04	2.28 (-0.33–4.88)	0.09
Diabetes	44.5 (16.7–72.3)	0.002	7.1 (-19.3-33-4)	0.6	0.67 (-1.53–2.87)	0.5	1.14 (-1.54–3.84)	0.4
Smoking	0.1 (-37.0–37.2)	1.0	6.7 (-24.0–37.5)	0.4	-1.19 (-3.97–1.60)	0.40	-2.56 (-5.70–0.58	0.1
Positive family history	10.4 (-15.8–36.7)	0.8	-1.20 (-22.3–19.9)	0.9	0.71 (-1.27–2.69)	0.5	0.99 (-1.17–3.14)	0.4

MV adjustment includes age, gender, BMI, systolic blood pressure, antihypertensive medication, LDL- and HDL-cholesterol, lipid-lowering medication, diabetes, smoking, positive family history.

### Association of EAT volume and CT-derived attenuation with clinical presentation

**Fig 1:**
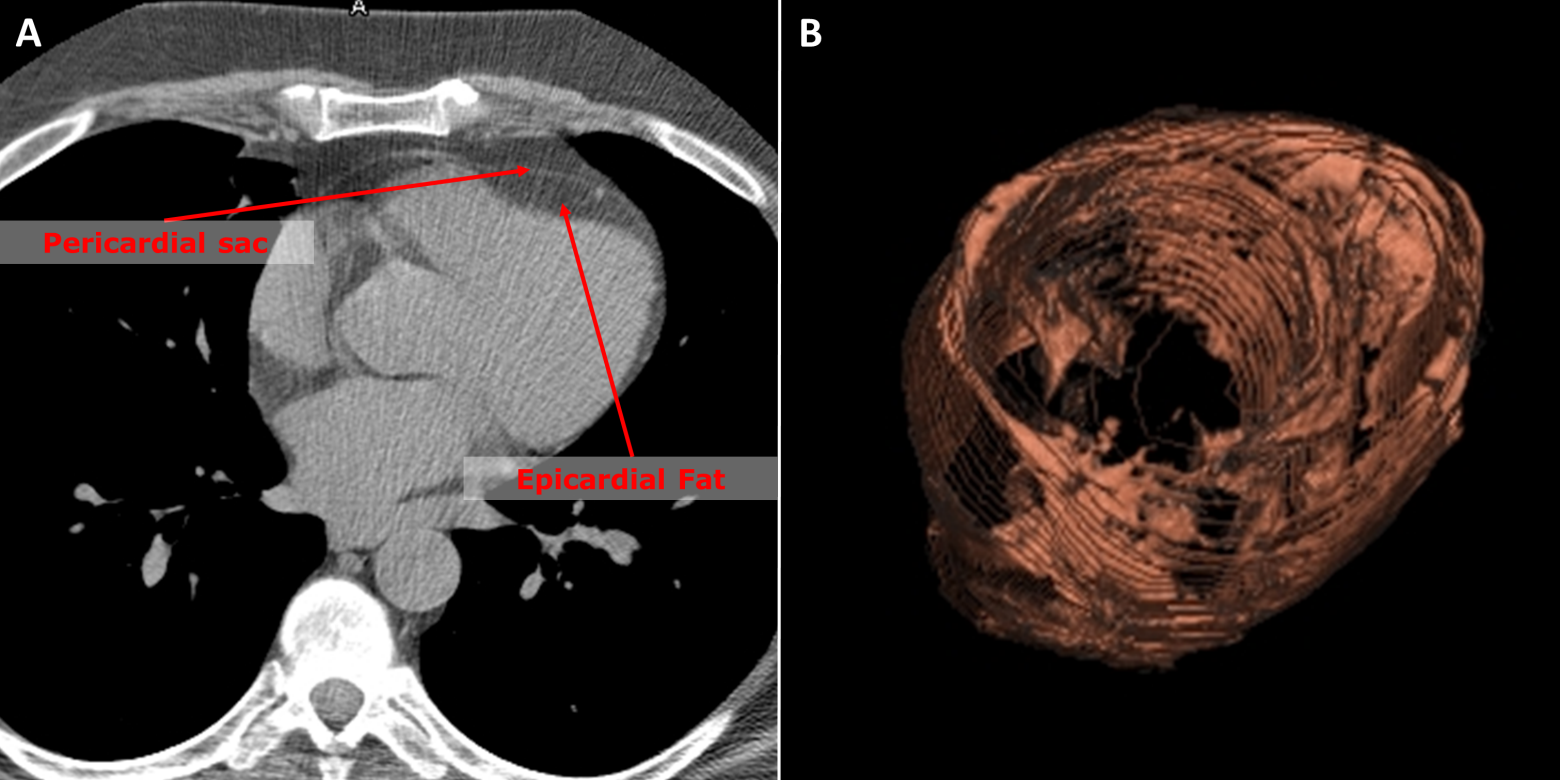
Epicardial fat quantification from cardiac CT examination. The pericardial sac was manually traced in axial images as region of interest (A). Within this region of interest, pixels between -195 and -45 Hounsfield Units were accounted as fat. After 3-dimensional reconstruction, EAT volume was calculated by summation of all pixels accounted as fat (B). EAT attenuation was defined as mean Hounsfield Units of all fat pixels of the EAT volume.

**Fig 2:**
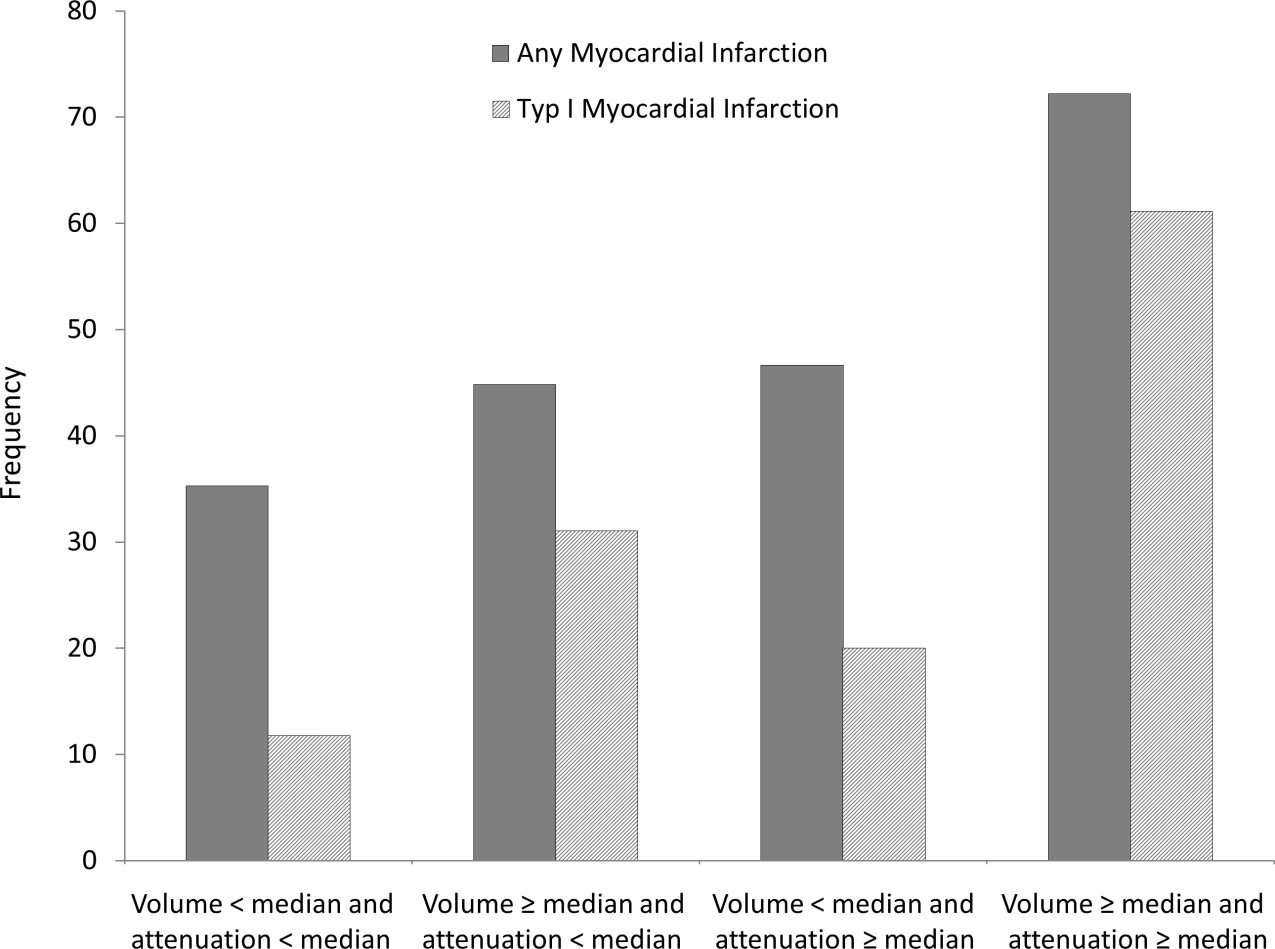
EAT volume and attenuation and any as well as type-I myocardial infarction. Frequencies of any (grey) and type-I myocardial infarction (dashed), stratified by combination of EAT volume and CT-derived attenuation above vs. below median, demonstrating the complementary value of EAT volume and attenuation.

Overall, 46 patients had a myocardial infarction. Of those, 28 had a type-I myocardial infarction due to coronary stenosis or occlusion, while 18 patients had a type-II myocardial infarction without a culprit coronary lesion. In patients with any myocardial infarction compared to stable CAD, EAT volume was slightly higher (123.1 (SD 68.1) ml vs. 110.4 (SD51.3) ml, p = 0.31). In contrast, EAT attenuation was significantly different between both groups (-87.1 (SD 5.2) HU vs. -89.5 (SD 3.4) HU, p = 0.01). Likewise, patients with type-I myocardial infarction had higher EAT attenuation (-86.8 (SD 5.8) HU vs. -89.0 (SD 3.7) HU, p = 0.03) and a tendency towards higher EAT volume (132.9 (SD 54.3) ml vs. 109.7 (SD 61.5) ml, p = 0.07) than subjects without type-I myocardial infarction. In logistic regression analysis, we observed robust positive associations of EAT volume with any myocardial infarction and type-I myocardial infarction in varying adjustment sets. However, despite stable effect sizes, no significant associations were observed in fully adjusted models (Table 3). Likewise, also EAT attenuation was positively associated with any and type-I myocardial infarction, independently of EAT volume. In contrast to EAT volume, association of EAT attenuation with any and type-I myocardial infarction remained stable and statistically significant upon adjustment for risk factors (Table 3). Interestingly, both EAT volume and EAT attenuation were associated with myocardial infarction when adjusting for each other. To further evaluate the potential complementary value of EAT volume and attenuation, we assessed the frequencies of acute coronary syndromes in our cohort stratified by EAT volume and attenuation below vs. above the median (Fig 1). Lowest frequencies of myocardial infarctions were observed for patients with EAT volume and attenuation below their median (any: 35.3%, type-I: 11.8%). Patients with diverse EAT profiles showed comparable frequencies of myocardial infarction (any: 44.8% vs. 46.7%, type-I: 31.0% vs. 20.0%, for volume above median and attenuation below median vs. volume below median and attenuation above median). The highest frequencies of myocardial infarction were observed for patients with both EAT volume and attenuation above their median (any: 72.2%, type-I: 61.1%, Fig 2).

**Table 3:** Association of EAT volume and EAT attenuation with any myocardial infarction and type-I myocardial infarction comparted to patients without myocardial infarction / without type-I myocardial infarction in crude adjusted and multivariable adjusted logistic regression analysis. OR are depicted per each standard deviation of EAT volume / attenuation.

	Model	Any myocardial infarction		Type-I myocardial infarction	
		OR (95% CI)	p-value	OR (95% CI)	p-value
**EAT volume**	EAT attenuation adjusted	1.54 (0.97–2.54)	0.07	1.79 (1.10–2.94)	0.02
	EAT attenuation, age, and gender adjusted	1.84 (1.08–3.12)	0.02	1.75 (1.03–2.96)	0.04
	+BMI and lipid-lowering medication adjusted	1.64 (0.88–3.07)	0.12	1.44 (0.77–2.68)	0.26
**EAT attenuation**	EAT volume adjusted	2.13 (1.25–3.64)	0.006	2.04 (1.18–3.53)	0.01
	EAT volume, age, and gender adjusted	2.11 (1.22–3.63)	0.007	2.00 (1.16–3.45)	0.01
	+BMI and lipid-lowering medication adjusted	2.17 (1.24–3.79)	0.007	1.93 (1.11–3.39)	0.02

## Discussion

We here show that (1) EAT volume and CT-derived attenuation are only modestly correlated while volume shows a wider range than attenuation, that (2) EAT attenuation increased with lipid-lowering medication, and that (3) CT-derived EAT attenuation is increased in patients with myocardial infarction, independently of EAT volume and risk factors. While associations of EAT attenuation with myocardial infarction remained stable and statistically significant even after adjustment for EAT volume and traditional risk factors, this was not the case for EAT volume, for which no significant associations were observed when controlling for EAT attenuation and risk factors. While this may at least in parts be explained by limited power, together with higher effect rates for EAT attenuation this finding suggests that EAT attenuation may more strongly reflect adverse characteristics of EAT compared to volume. Our results suggest that quantification of CT-derived EAT attenuation may contain complementary information to EAT volume which may increase the clinical value of quantification of epicardial fat from cardiac CT images.

The association of EAT volume with coronary artery plaque burden and coronary artery disease is well documented in the literature. Increased EAT volume is not only associated with calcified and non-calcified coronary plaque burden,[1, 2, 4] but also linked with high risk plaque characteristics.[15] As EAT volume is associated with incident myocardial infarction, its assessment increases the prognostic value of non-contrast cardiac CT imaging above CAC-scoring.[3, 16] In addition to EAT volume, also its CT-derived attenuation has recently gained interest as it increases with vascularization and concentration of mitochondria and decreases with overload of fatty acids.[7] CT derived fat attenuation is correlated with local and systemic inflammatory markers and therefore is suggested to reflect unfavorable metabolic activity.[8, 9] EAT modulates local inflammation in the immediate surrounding of the coronary arteries, and an increase in pro-inflammatory mediators and cytokines in the EAT was observed in subjects with coronary artery disease when undergoing open heart surgery. [17–23] With increasing EAT volume, the amount of adiponectin, a stabilizer of the inhibitor of NFκB released from pericardial fat, decreases.[20, 24] In addition, epicardial fat contains neural structures innervating the myocardium and vessels. Therefore, the influence of neural structures on myocardial infarction may be mediated via epicardial fat. Changes in parasympathetic innervation alter vasomotor function, cAMP levels and ventricular refractory periods.[25] This may lead to increased risk of not only vascular events but also atrial and ventricular arrhythmias, which are also associated with epicardial fat volume.[12, 25, 26].

In a recent case-control study on subjects undergoing myocardial perfusion imaging, EAT volume but not attenuation was associated with myocardial ischemia. However, this analysis was based on a cohort of subjects with suspected stable CAD,[11] while we investigated CT-derived EAT attenuation in patients with and without myocardial infarctions. Further, fat attenuation was found to be correlated with activated conditions of adipose tissue, as reflected by an increase in FDG-uptake in PET/CT studies.[27] These findings are supported by our results, suggesting that an activation of EAT metabolic activity could lead to an increase in CT-attenuation. We also found higher EAT attenuation in patients with statin therapy, which were more frequently on lipid lowering medication. Therefore, an influence of statin therapy on EAT attenuation may have influenced the observed association of EAT attenuation with myocardial infarction. Further studies comparing EAT attenuation with local and systemic metabolic and inflammatory profiles are needed to understand underlying mechanisms. Besides overall epicardial adipose tissue volume, also the location of fat may relevantly contribute to a potential link with coronary artery disease. Peri-coronary fat volume surrounding coronary segments with present plaque burden is higher compared to segments without plaque burden and is increased in segments in which acute coronary syndromes occur even years later, suggesting that a local association of peri-coronary fat with plaque development exists.[5] Further studies are needed to determine, whether CT-derived fat attenuation is also associated with plaque burden on a coronary segment level.

### Limitations

Overall, our analysis is limited by sample size which has ultimately restricted our statistical power. However, despite small sample size, we observed stable and significant associations which may underline the potential value of assessment of EAT attenuation. Further studies in larger cohorts need to confirm our results. Most importantly, we were limited by the number of patients who underwent CT imaging during hospitalization for myocardial infarction, since in patients with myocardial infarction, cardiac CT is limited to rare indications. Also, we randomly selected an equal amount of subjects without myocardial infarction. Therefore, our results may not represent the cohort of patients undergoing cardiac CT in clinical routine as well as typical patients with myocardial infarction. Also, we can only hypothesize the pathophysiological link between CT-derived fat attenuation and coronary artery disease, which needs to be evaluated in dedicated studies. Lastly, our data are based on a cross-sectional evaluation; hence conclusions regarding causality cannot be made.

## Conclusion

CT-derived EAT attenuation distinguishes patients with vs. without myocardial infarction over and above EAT volume and is increased in patients with lipid-lowering therapy. Our results suggest that assessment of EAT attenuation could render complementary information to EAT volume regarding coronary risk burden.

## Supporting information

S1 Fig Scatter plot for the correlation of EAT volume and CT-derived attenuation. EAT volume and attenuation show a modest negative correlation (r = -0.24, p = 0.02).
(DOCX)

## Abbreviations

BMI: body mass index
CI: confidence interval
CT: computed tomography
EAT: epicardial adipose tissue
HDL: high density lipoprotein
HU: Hounsfield units
IQR: interquartile range
LDL: low density lipoprotein
MV: multivariable
OR: odds ratio
SD: standard deviation
