# Supplementary material for: Cardiac computed tomography-derived epicardial fat volume and attenuation independently distinguish patients with and without myocardial infarction
Source: PLoS One. 2017 Aug 24;12(8):e0183514. doi: 10.1371/journal.pone.0183514 (PMC5570500; doi:10.1371/journal.pone.0183514)

**S1 Fig. Scatter plot for the correlation of EAT volume and CT-derived attenuation.** EAT volume and attenuation show a modest negative correlation (r=-0.24, p=0.02).


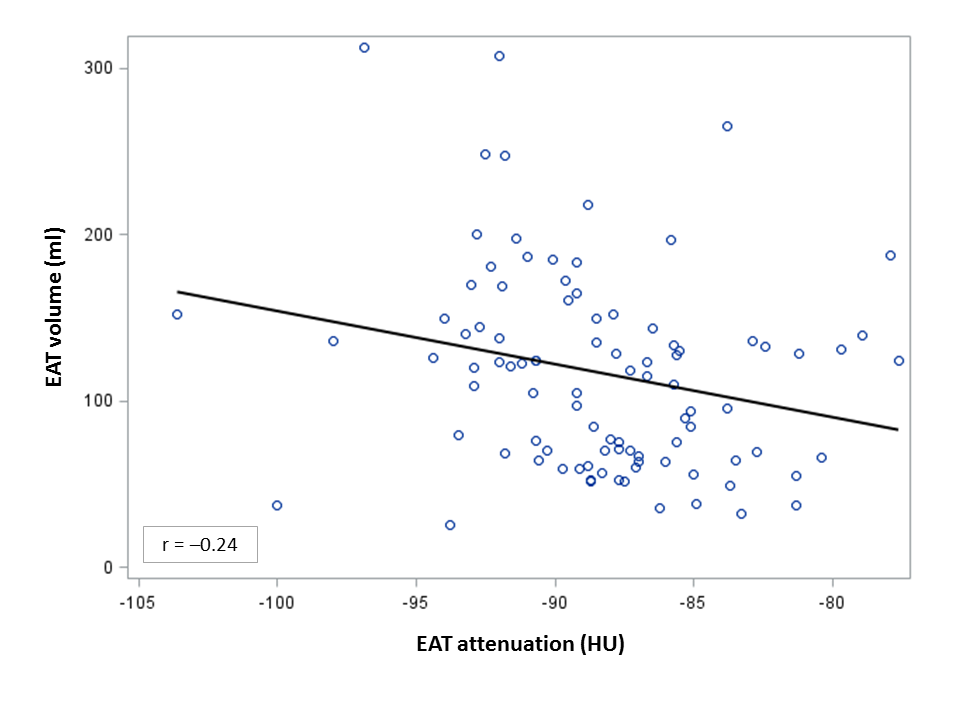

Supplement: S1 Fig — EAT volume and attenuation show a modest negative correlation (r = -0.24, p = 0.02). (DOCX) [file pone.0183514.s001.docx]
